# A Multidisciplinary, Family-Oriented Approach to Caring for Parents After Miscarriage: The Integrated Behavioral Health Model of Care

**DOI:** 10.3389/fpubh.2021.725762

**Published:** 2021-11-30

**Authors:** Angela R. Hiefner, Astrud Villareal

**Affiliations:** Family and Community Medicine, University of Texas Southwestern Medical Center, Dallas, TX, United States

**Keywords:** miscarriage, pregnancy loss, perinatal loss, biopsychosocial, integrated care, family-oriented approach, multidisciplinary approach, primary care behavioral health integration

## Abstract

Miscarriage is increasingly gaining recognition, both in scientific literature and media outlets, as a loss that has significant and lasting effects on parents, though often disenfranchised and overlooked by both personal support networks and healthcare providers. For both men and women, miscarriage can usher in intense grief, despair, and difficulty coping, and for women in particular, there is evidence of increased prevalence of depression, anxiety, and post-traumatic stress. Additionally, miscarriage can contribute to decreased relationship satisfaction and increased risk of separation, all while stigma and disenfranchisement create a sense of isolation. Despite this increased need for support, research indicates that many parents experience their healthcare providers as dismissive of the significance of the loss and as primarily focusing only on the physical elements of care. Research exploring the barriers to providers engaging in more biopsychosocial-oriented care has identified time constraints, lack of resources, lack of training in addressing loss, and compassion fatigue as key areas for intervention. This paper will review the biopsychosocial elements of miscarriage and discuss a multidisciplinary, family-oriented approach that can be implemented in healthcare settings to ensure a high quality and holistic level of care for individuals, couples, and families experiencing pregnancy loss.

## Introduction

Miscarriage is a medical event with a complex combination of psychosocial sequelae, however research indicates that healthcare providers and clinical teams often fail to attend to the complex and sensitive nature of miscarriage (1, 2). For many parents, miscarriage is a traumatic loss, but not always recognized as such by important sources of support in their social and healthcare networks (1–4). This paper will review the biopsychosocial elements of miscarriage, discuss barriers to biopsychosocial approaches to miscarriage care, and propose a family-oriented, multidisciplinary approach that can address these barriers and provide parents with holistic, sensitive care after their loss.

## A Biopsychosocial Understanding of Miscarriage

### Biological

Miscarriage is more common than people often believe (5), occurring in about 31% of all pregnancies, though a portion of these occur prior to a woman's knowledge of her pregnancy (6, 7). In clinically diagnosed pregnancies, about 8–15% end in miscarriage (8, 9). Miscarriage, early pregnancy loss, and spontaneous abortion are all terms that are used interchangeably to describe the loss of a pregnancy during the first 20 weeks (10). Miscarriage and the resulting experience of loss are distinct from other perinatal losses, such as stillbirth, which is the death of a fetus after 20 weeks' gestation, or elective abortions that surgically or medically end a pregnancy prior to fetal viability (11).

There are several risk factors associated with miscarriage, including advanced maternal age, certain medications, maternal infections, and previous miscarriage (9). However, the majority of miscarriages do not have a known cause, and this can create additional challenges as parents attempt to understand what has happened, cope with the loss, and plan for future pregnancies (12). A large number of myths exist regarding other contributing factors for miscarriage (e.g., air travel, sexual activity, a prior elective abortion), though these have garnered no scientific evidence of increased risk of miscarriage (9, 13). Once a miscarriage has occurred, some biological factors are also associated with worse psychological outcomes, including older maternal age (14), history of infertility (15), unknown cause of the pregnancy loss (16), and recurrent miscarriages (17).

Previously, most miscarriages were managed in the hospital setting, and though these patients continue to be cared for in emergency departments and on labor and delivery floors, present-day miscarriage management now occurs more frequently in the outpatient setting with a patient's primary care physician or OB/GYN (9). There are three management strategies: (1) expectant management, in which the body manages the loss on its own, (2) medical management, in which the patient is sent home with medications to aid the miscarriage process, and (3) surgical management, in which the miscarried pregnancy is surgically removed. Choosing between these management options can be a difficult decision for patients, and provision of information, opportunity to ask questions, and assurance that this choice will not affect future fertility are important elements of care for these patients (9, 18, 19).

### Psychosocial

Though often perceived as a loss primarily impacting women (and for lesbian partners, the partner who carried the pregnancy), miscarriage impacts both partners in a relationship (20–22), and even other family members, as well (23). For both men and women, miscarriage can usher in intense grief, despair, and difficulty coping (24), and for women in particular, there is evidence of increased prevalence of depression, anxiety, and post-traumatic stress (25, 26). Positive social support, a satisfying partner relationship, and already having a child are protective factors against depression and anxiety after this loss (27).

Though grief has been shown to decrease over a 4 month period for both genders, isolation, feelings of loss, and the perception of the loss as a devastating event can persist over time (24). Many women may also place blame on themselves for the loss, experiencing significant guilt and feelings of failure as a woman or as a mother (28). Grandparents of the baby may experience grief as well, and the experience of seeing their own child grieve can add complexity to that loss (29). Siblings are an additional group that may struggle, sometimes invisibly, with miscarriage; as parents attempt to cope, siblings' questions and feelings of loss may inadvertently be overlooked (30). Each of these grief experiences can be exacerbated by the disenfranchisement of this loss.

Disenfranchisement is a key element in a biopsychosocial understanding of miscarriage. A disenfranchised loss is a loss that is “not openly acknowledged, publicly mourned, or socially supported” [(31), p. 4]. A growing body of research points to disenfranchisement as an aspect of miscarriage that impedes parents' abilities to successfully grieve and cope with their loss (2–4, 32). Though social support is a critical factor in bereavement outcomes (33), family members, friends, healthcare providers, and even society more generally often fail to understand and validate the meaning and significance of miscarriage loss. The lack of understanding about grief after miscarriage is pervasive, and likely perpetuated by the norms of silence surrounding early pregnancy and pregnancy loss (34).

Though often well-intentioned, many family members and friends make statements that minimize the loss (e.g., “You can always have another,” “At least you know you can get pregnant”), resulting in bereaved parents feeling they do not have permission or space to experience and express their grief (2). Medical providers across multiple specialties (particularly OB/GYN, primary care, and emergency) regularly care for parents experiencing miscarriage, however, research indicates that bereaved parents are infrequently asked how they are coping after a miscarriage and often experience their providers as dismissive of the loss, which has been shown to increase women's distress (1, 4).

In addition to impacts on individual partners, research shows that miscarriage can also significantly impact the couple relationship. During a time when stigma and disenfranchisement can create a sense of isolation for one or both partners (3), miscarriage is also associated with decreased relationship satisfaction (35, 36) and increased risk of separation (37), further compounding the stress and difficulty coping parents may experience after their loss. Research indicates that these relational impacts result from high levels of distress (38), differing perceptions of the meaning of the loss (4), incongruences in expression of grief and desired support (36, 37), avoidance coping strategies that reduce emotional support within the relationship (35), and even different expectations between partners regarding how to react to the loss and how to grieve (4, 35). However, despite these challenges, some couples experience relationship growth after miscarriage as a result of turning toward each other for support during a difficult time, embracing both similarities and differences in their grief, and experiencing support and care from their partner (39, 40). Partners experiencing growth after miscarriage cite availability of and quality of support as important factors enabling this growth (39). Enhancing support across both personal and healthcare networks may improve parents' abilities to cope with this difficult loss, and perhaps even contribute to reductions in the level of disenfranchisement accompanying miscarriage.

## Current Challenges in Miscarriage Care

Healthcare providers are frequently a patient's first point of contact during or after miscarriage as they experience concerning symptoms, seek help, and receive a diagnosis, or as they follow up with their provider and discover their baby is no longer growing as expected. As this first point of contact, healthcare providers are a crucial first step in supporting parents as they navigate this loss. However, research has consistently documented significant gaps in the psychosocial elements of miscarriage care, including lack of empathy, treating miscarriage as routine and trivial, failing to attend to grief and loss, and lack of clarity in communication about the miscarriage and next steps (1, 4, 41–44).

A recent study by Jensen et al. (45) investigated healthcare providers' experiences, and found that these limitations in care are largely due to a lack of training in managing the psychosocial aspects of miscarriage, limited time, inadequate resources, and compassion fatigue. Additionally, many medical schools and residency programs lack a strong emphasis even in the medical management of miscarriage, beyond expectant management (46–48), which may reduce healthcare providers' abilities to engage patients in shared decision-making regarding miscarriage management, an important element of care associated with patient satisfaction (49). Though many healthcare providers would like to provide biopsychosocial-oriented care, they simply lack key resources to do so.

## A Family-Oriented, Multidisciplinary Approach to Miscarriage Care

In light of the overwhelming amount of evidence indicating the sub-par quality of existing approaches to miscarriage care, researchers are calling for new methods of care and new interdisciplinary team members to improve care across all levels of the biopsychosocial spectrum (42, 50). Current recommendations for enhancing psychosocially-oriented, patient-centered care include: (1) attending to the emotional significance of the loss, (2) providing more information to parents regarding miscarriage management and impact on fertility, (3) engaging patients and their partners in shared decision-making, (4) implementing screening to identify needs for additional mental health support and (5) developing a referral system and resource list to connect parents with this support (1, 50–53). DiMarco et al. (54) have also recommended the implementation of educational programs to build healthcare providers' expertise in delivering this kind of supportive miscarriage care. To address these recommendations, we suggest three key strategies for implementation of a family-oriented biopsychosocial approach to miscarriage care that can facilitate these important action items while simultaneously addressing the barriers that impede their use (e.g., time constraints, lack of resources, compassion fatigue).

### Establish a Multidisciplinary Team

The integrated behavioral health (IBH) model of clinical practice is an innovative and multidisciplinary approach to care that can address the barriers to high quality miscarriage care and enable healthcare practices to implement these care recommendations. In the IBH model, behavioral health providers (BHPs) are hired by the clinic, creating a multidisciplinary team able to address both biological and psychosocial elements of miscarriage under one roof (55). These clinicians come from a variety of professional backgrounds, including marriage and family therapy, professional counseling, clinical social work, and psychology. The care team members in these integrated clinics work side by side and within the same electronic health system to enable collaborative, team-based care (56).

To adapt to the healthcare setting, BHPs in these practices conduct appointments that range from 15 to 30 minutes, while also maintaining flexibility in order to be available for consultations with physician and nurse team members (55). During these consultations, the care team may decide to coordinate a “warm handoff” to connect a patient to a BHP. In a warm handoff, a physician introduces the patient to the BHP during the patient's medical visit, creating space for the BHP to establish rapport, as well as conduct a brief intervention and/or discuss treatment options (57). Though most frequently implemented in primary care, this model can also be adapted for other specialties that regularly care for patients experiencing miscarriage, such as emergency departments and outpatient OB/GYN clinics (58, 59).

### Develop a Miscarriage Protocol

Through this collaborative approach, the care team shares responsibility for each patient's well-being. Clinical settings using this model of practice often develop clinical protocols for specific diagnoses or conditions for which a BHP is regularly involved (55). In these types of protocols, the clinic's BHP is automatically connected with patients who meet specific criteria (e.g., diabetes diagnosis, positive depression screening, smoking cessation counseling). Miscarriage can be included in these protocols, establishing a behavioral health warm handoff as a regular part of miscarriage care in that clinic. This warm handoff can include an assessment of how the patient and their family are coping, create space for empathy and validation of the loss, offer psychoeducation regarding grief after miscarriage, and discuss what support and resources are available to them.

During the initial assessment, the BHP works collaboratively with the patient and family to discuss support needs and follow up options. Subsequent to the initial warm handoff, the BHP schedules follow up appointments based on each patient and family's treatment needs. For some patients, helpful follow up options may also include connection to pregnancy loss support groups, pastoral or spiritual support, and additional fertility information from their medical provider (e.g., fertility treatment options, genetic counseling). In the IBH model, BHPs and medical providers work collaboratively in the treatment of each patient, communicating about clinical assessments, treatment goals, and progress.

By implementing a multidisciplinary care team and standard involvement of a BHP for all patients experiencing miscarriage, healthcare teams can improve the quality of care patients receive by increasing access to psychosocial care and reducing the amount of care burden that falls to physician team members. Though not all patients will require the same level of support, all patients will know this support is accessible to them if needed. When physicians are no longer tasked with the impossible job of caring for all elements of a patient's health in a small window of time, they may experience reduced stress levels and feel more freedom to engage with the psychosocial elements of miscarriage care knowing they have a team member with whom they can connect their patient (60). This shared-care protocol may also create more space for shared decision-making regarding the medical management of miscarriage, as well as more time for physicians to address patients' concerns about future fertility.

### Consider Family

Because miscarriage is often viewed primarily as an issue affecting mothers, other family members struggling with the loss may be overlooked. A growing literature base is identifying fathers' needs for support after miscarriage (61), and grandparents and siblings of the baby may also benefit from support as they navigate what the loss means for them (62, 63). With this type of loss often unacknowledged or misunderstood for mothers, other family members' grief may be even more invisible. Additionally, miscarriage can create stress in partner and family relationships as individuals cope in different ways and struggle to navigate the loss together (35, 39).

Miscarriage's broad impact on multiple family members, as well as on the relationships between partners and family members, highlights the need for care that is not only biopsychosocial, but also family-oriented. Clinicians working with women experiencing miscarriage can expand their assessment to include questions about the patient's support system and how those individuals are responding to the loss. This practice can increase the amount of support a family receives through opportunity to connect family members to behavioral health services, as well as offer other miscarriage support resources. BHPs can invite partners and family members to participate in the behavioral health services they provide their patients. This couple and family level of care can support family members in exploring their unique experiences of the loss, meanings of the loss, issues related to identity and guilt, expression of grief, grieving together and separately, emotional intimacy after loss, physical intimacy after loss, and shared experiences of disenfranchisement (3, 28, 36, 39). Additionally, for primary care practices, the patient may be a partner or family member of someone who has miscarried; as part of a biopsychosocial approach applied to all patients, providers may discover the impact of miscarriage while treating these patients and have an opportunity to mobilize the clinic's additional resources for them as well.

## Conclusion

Though there is extensive research on psychological outcomes after miscarriage, primarily for women, there remain significant gaps in the literature base regarding a family-oriented understanding of the experience of miscarriage, family level grief outcomes and relational impacts, and biopsychosocial-oriented healthcare for patients and families facing this loss. Additionally, research has not yet tested the IBH model in miscarriage care. As an existing, evidence-based model of care (64, 65), IBH represents an important opportunity to address the limitations of current miscarriage care, as well as the barriers to implementation of family-oriented, biopsychosocial care (1, 41, 44).

As many patients' first point of care for miscarriage, healthcare providers are in a unique position to positively influence these patients' loss experiences. Empathic, biopsychosocial care can set a trajectory for successful coping and sufficient support, particularly during an experience that is often disenfranchised. By implementing an integrated behavioral health model of care, creating a protocol, and considering patients' larger familial context, healthcare providers can increase the amount of support and resources available to bereaved parents and their families.

## Data Availability Statement

The original contributions presented in the study are included in the article/supplementary material, further inquiries can be directed to the corresponding author.

## Author Contributions

AH led the conception and outline of this manuscript. AH and AV contributed as co-authors to the full draft of the manuscript. All authors contributed to the article and approved the submitted version.

## Conflict of Interest

The authors declare that the research was conducted in the absence of any commercial or financial relationships that could be construed as a potential conflict of interest.

## Publisher's Note

All claims expressed in this article are solely those of the authors and do not necessarily represent those of their affiliated organizations, or those of the publisher, the editors and the reviewers. Any product that may be evaluated in this article, or claim that may be made by its manufacturer, is not guaranteed or endorsed by the publisher.
